# Study on Photoelectric Properties and Applications of Nanostructured Materials

**DOI:** 10.3390/nano16191252

**Published:** 2026-10-03

**Authors:** Yunqing Tang, Bing Yang, Yanfang Zhao, Ping Yang

**Affiliations:** 1 School of Mechanical Engineering, Shandong University, Jinan 250061, China; 2 State Key Laboratory of Advanced Equipment and Technology for Metal Forming, Shandong University, Jinan 250061, China; 3 Key Laboratory of High Efficiency and Clean Mechanical Manufacture of Ministry of Education, Jinan 250061, China; 4 Centre for Advanced Laser Manufacturing (CALM), School of Mechanical Engineering, Shandong University of Technology, Zibo 255000, China; 5 School of Mechanical Engineering, Jiangsu University of Technology, Changzhou 213001, China; 6 Laboratory of Advanced Design, Manufacturing & Reliability for MEMS/NEMS/OEDS, School of Mechanical Engineering, Jiangsu University, Zhenjiang 212013, China

## 1. Introduction

Nanostructured materials offer many ways to tailor light absorption, charge transport, and light emission because these responses are sensitive to composition, dimensionality, and local structure. Graphene-like ZnO is one example: first-principles studies have examined its electronic and optical properties [1] and shown how substitutional doping changes its electronic states [2,3]. Optical response can also be modified without changing the host composition. Surface-plasmon coupling, for instance, has been used to enhance emission from InGaN quantum wells and ZnO thin films [4,5,6].

What constitutes an effective design depends on the application. Light-emitting devices require efficient radiative recombination and light extraction, whereas photocatalysts depend on charge separation and transfer to support surface reactions. In infrared and terahertz detection, photodetectors integrated with light-emitting diodes can convert long-wavelength radiation into near-infrared emission [7,8,9]. TiO_2_/graphene systems provide another example of this application-specific behavior: electron transfer between the components [10], improved photocatalytic activity [11], and the role of interfacial bonding in charge transfer [12] have all been investigated. Graphene, MoS_2_, and GeTe have also been used as saturable absorbers for passive mode locking in fiber lasers [13,14,15].

Theory is particularly useful in regard to these systems when it helps explain why a measured response changes. Combined first-principles calculations and experiments on g-C_3_N_4_/ZnO heterojunctions have linked interfacial configuration and charge redistribution with photocatalytic activity [16]. Calculations on MoS_2_/MoSe_2_ van der Waals heterojunctions have examined band alignment, charge separation, and optical absorption for photocatalytic water splitting [17], while density functional theory has been used to evaluate structural stability, electronic structure, phonon behavior, and optical response in two-dimensional metals [18]. Such studies provide an atomic-scale basis for interpreting the structure–property relationships observed experimentally.

This Special Issue, “Study on Photoelectric Properties and Applications of Nanostructured Materials,” brings together five research articles that approach these questions from different directions. They address doped graphene-like ZnO, plasmon-enhanced ZnO emission, cryogenic infrared light-emitting diodes for broadband upconversion, GO/TiO_2_ photocatalysts, and GeTe saturable absorbers. The materials and devices are different, but each contribution examines how composition, interfaces, or nanoscale structure can be used to control a specific optical or photoelectric process.

## 2. Overview of the Contributions

Bao et al. [19] examined Li, Ag, and F doping in graphene-like zinc oxide (g-ZnO) using first-principles calculations. Individual Li or Ag substitution produced shallow acceptor levels, whereas Li-F co-doping in a 1:1 ratio resulted in donor–acceptor compensation and no acceptor levels. Configurations containing two acceptor dopants and one F donor again showed shallow acceptor states. Among the structures considered, g-Zn_14_Li_2_FO_15_ displayed a strong absorption peak near 520 nm. This work therefore identifies several possible routes toward achieving p-type g-ZnO while leaving the predicted carrier behavior to be tested experimentally.

Tokimori et al. [20] took a different route to modifying ZnO emission. They deposited ZnO thin films on structures composed of Au nano-hemispheres, an Al_2_O_3_ spacer, and an Al mirror and tuned the localized surface plasmon resonance by varying spacer thickness. At approximately 380 nm, the ZnO photoluminescence peak intensity was 35 times that of a reference ZnO film on sapphire annealed at the same temperature, 630 °C. Temperature-dependent measurements also indicated an increase in internal quantum efficiency. These results show how the optical environment around ZnO can be engineered to enhance emission without altering the emitting layer itself.

At the device level, Bai et al. [21] investigated GaAs-based infrared light-emitting diodes for semiconductor broadband upconversion under cryogenic conditions and weak current injection. An optimized heterojunction was combined with surface microstructures to improve carrier injection and light extraction. Below 20 K, the microstructured devices showed approximately 35% higher electroluminescence efficiency than planar devices, although the advantage decreased as temperature increased. The authors related this behavior to stronger non-radiative recombination, including surface recombination. The optimized devices also produced more uniform emission than the earlier unoptimized structure.

Duan et al. [22] focused on interfacial charge transfer in GO/TiO_2_ photocatalysts. Microwave treatment at 600 W for 5 min resulted in the highest activity for Rhodamine B degradation, with a pseudo-first-order rate constant of 0.028 min^−1^, which is about four times that of pure TiO_2_. Relative to the untreated composite, the optimally treated material showed less particle agglomeration, a larger specific surface area, and optical absorption extending to longer wavelengths. Transient photocurrent and electrochemical impedance measurements were also consistent with improved charge separation and interfacial transfer. The performance increase thus reflects several structural and electronic changes acting together.

Tang et al. [23] explored a different application of nanostructured materials by preparing GeTe nanosheets through liquid-phase exfoliation and incorporating them into a polyvinyl alcohol film as a saturable absorber for an erbium-doped fiber laser. The film had a modulation depth of 8% and a saturation intensity of 13.7 MW/cm^2^. With an output coupling ratio of 80%, the laser reached a single-pulse energy of 32 nJ and an average output power of 50.48 mW at a pump power of 450.1 mW, corresponding to an optical-to-optical conversion efficiency of 11.2%. The results also show that the output coupling ratio affects both pulse energy and conversion efficiency, linking material response with laser-cavity design.

## 3. Conclusions and Outlook

The five contributions illustrate that improvements in photoelectric performance arise from different physical processes and therefore require different design strategies. Doping of g-ZnO changes electronic states; plasmonic structures modify the local optical environment; surface microstructures in infrared light-emitting diodes influence carrier injection and light extraction; microwave treatment of GO/TiO_2_ affects morphology and interfacial charge transfer; and GeTe acts through nonlinear optical absorption in a laser cavity. Several issues remain to be resolved before these approaches can be assessed under practical conditions. For doped g-ZnO, carrier type and concentration should be measured experimentally to determine whether the calculated acceptor states produce stable p-type conductivity. For light-emitting structures, surface passivation and optical absorption loss deserve attention, especially over the intended operating-temperature range. Repeated-use tests and experiments in real wastewater would provide a more demanding assessment of GO/TiO_2_ photocatalysts, while long-term operation and pulse splitting at higher pump power remain relevant for GeTe-based lasers. Reproducible preparation and clearly defined testing conditions are essential for meaningful comparison.

A further consideration is that the same interfaces used to control charge and optical processes must also remain structurally and thermally reliable. Studies on graphene/Si and graphene/SiC heterogeneous interfaces have shown that interfacial bonding configuration and bonding density affect interfacial strength and failure behavior and that suitable thermal treatment can improve interfacial bonding and thermal reliability [24]. Interface morphology is equally important for heat transfer: nanoscale nonplanar features in w-AlN/graphene/3C-SiC heterostructures can alter interfacial thermal resistance through changes in interfacial interactions and phonon density-of-states overlap [25]. At smaller scales, phonon mismatch and localization at graphene-related interfaces or boundaries can impede heat transport [26,27], whereas interfacial reconstruction or optimized interfacial superlattice nanostructures can strengthen phonon coupling and increase thermal conductance [28,29]. These effects are directly relevant to temperature-sensitive optoelectronic devices, including the cryogenic infrared light-emitting diodes reported in this Special Issue [21]. Future interface design should therefore consider electronic or optical function together with mechanical stability and heat dissipation rather than treating them as independent requirements.
